# Validity of a Self-administered Food Frequency Questionnaire Used in the 5-year Follow-up Survey of the JPHC Study Cohort I to Assess Fatty Acid Intake: Comparison with Dietary Records and Serum Phospholipid Level

**DOI:** 10.2188/jea.13.1sup_64

**Published:** 2007-11-30

**Authors:** Minatsu Kobayashi, Satoshi Sasaki, Terue Kawabata, Kyoko Hasegawa, Shoichiro Tsugane

**Affiliations:** 1 Epidemiology and Biostatistics Division, National Cancer Center Research Institute East.; 2 Division of Basic Nutrition, Kagawa Nutrition University, Sakado, Japan.

**Keywords:** fatty acids, validity, food frequency questionnaire, dietary record, serum phospholipid

## Abstract

We compared fatty acid intake estimated from our 138-item food frequency questionnaire (FFQ) with 28-day weighed dietary records among a subgroup of JPHC Study Cohort I (102 men and 113 women), and with the corresponding two serum phospholipid levels (88 men). Spearman rank correlation coefficients between fatty acid intakes estimated from FFQ and intakes estimated from DR were as follows: saturated fatty acid, r=0.61 and r=0.60; monounsaturated fatty acid, r=0.50 and r=0.44; for energy adjusted value and Eicosapentaenoic acid (EPA), r=0.62 and r=0.55; docosahexaenoic acid (DHA), r=0.61 and r=0.50; for percentage of total fatty acid intake in men and women, respectively. Spearman rank correlation coefficients between fatty acid intakes estimated from FFQ and the corresponding serum phospholipid levels (% of total fatty acid) were as follows: EPA, r=0.43 and r=0.59; DHA, r=0.35 and r=0.49; for crude value (g/day) and percentage of total fatty acid intake, respectively. In conclusion, relatively high correlations were observed for SFA, MUFA and marine-origin n-3 polyunsaturated fatty acid, whereas we must take into account the indicator of each fatty acid intake when using the data of fatty acid intake assessed with FFQ for JPHC study.

The relationship between dietary intake of specific fatty acids and chronic disease has hardly been investigated in epidemiological studies. Several reports have shown a potential role for n-3 polyunsaturated acids (PUFA) in reducing the risk of chronic disease such as cancer and cardiovascular disease.^1^

Japanese people consume much more n-3 PUFA derived from fish than Western populations, and there is a greater variation of n-3 PUFA intake in Japanese diet.^2^ Also, Japanese people consume less saturated fatty acids (SFA) than Western populations.^3^^,^^4^ It is important to investigate the relationship between specific fatty acid intake and health effects in these populations. Therefore, it is essential to assess fatty acid intakes in a large population.

In the present study, we compared fatty acid intake estimated with our 138-item food frequency questionnaire with 28-day weighed dietary records among a subgroup of JPHC Study Cohort I. We also evaluated whether there was any relation between fatty acid intake and the fatty acid composition of serum phospholipid.

## MATERIALS AND METHODS

The study design and subject characteristics have been reported elsewhere in the present study.^5^ The survey method of dietary records (DR) and the computation method of nutrient intakes from the food frequency questionnaire (FFQ) have also been described elsewhere in this Supplement.^6^^,^^7^

### Fatty Acid Intake Calculations

The daily intake of fatty acids was calculated using the fatty acid composition table of Japanese foods.^8^ The table has missing values for some foods, so we substituted fatty acid composition for the missing foods.^9^

### Serum Phospholipid Fatty Acid

Peripheral venous blood was sampled just before 7-day DR in February and just after those in August 1994 in the Ninohe, Yokote and Saku areas. Blood was collected in the Ishikawa area in February and August 1995 under similar conditions. After leaving the blood for an hour at room temperature to facilitate clotting, the serum was separated by centrifugation. Serum was kept frozen in an icebox with sufficient dry ice until sent to the laboratories and stored at -80°C until analysis.

Serum phospholipid fatty acids were analyzed at the Division of Basic Nutrition, Kagawa Nutrition University. Serum lipids were extracted with dichloromethane/methanol (2/1; vol/vol). The phospholipid fraction was separated by thin-layer chromatography using a silica-gel plate and a solvent system of petroleum ether/ethyl ether/acetic acid (82:18:1, vol/vol/vol). Each phospholipid sample was then esterified in the presence of methanolic HCl and analyzed by gas chromatography (G-5000, Hitachi) equipped with a 0.25 mm×30 m capillary column (DB-225 J&B Scientific). The initial oven temperature was 140°C, and the ratio of increases was 2°C/min until the final temperature (220°C) was reached. Helium was used as the carrier gas with a flow rate of 30 mL/min. The temperatures at the injection port and the hydrogen flame ionization detector were 260°C. The identity of 31 individual FA was ascertained by comparing each peak's retention time with the retention times of synthetic standards of known FA compositions. The relative amount of each FA (% of total FAs) was calculated by dividing each FA by the total area for all FAs.

### Statistical Analyses

The 215 subjects (102 men and 113 women) both with FFQ for the validation study and the complete DR (14-day records in Okinawa and 28-day records in the other 3 areas) were included in this analysis. Eighty-eight men who provided blood samples both in Feb. and in August were included in the analysis using serum phospholipid.

All data e presented as mean values, SD and median each sex and area. Spearman rank correlation was used to assess the relations among estimated fatty acid intake from FFQ, DR, and serum phospholipid. For each fatty acid, we expressed the crude value (g/day), the energy-adjusted value by a residual model and the percentage of total fatty acid intake. To investigate the misclassification of subjects into the quintiles of fatty acid intake estimated from FFQ, they are classified against the quintiles according to both the fatty acid intake estimated from DR and serum phospholipid fatty acid level. All statistical analyses were performed using the SAS statistical software package (release 6.12, SAS Institute Inc.^10^

## RESULTS

Table 1 shows the intake levels of fatty acid by sex and area estimated from DR. In men, the mean total fatty acid intake, SFA intake, and monounsaturated fatty acid (MUFA) intake were lowest in Akita. In women, SFA intake was highest in Okinawa. Both in men and women, marine-origin n-3 fatty acid intake was lowest and the ratio of n-3 fatty acid intake to n-6 fatty acid intake was highest in Okinawa.

**Table 1.** Fatty acid intakes (g/day) assessed with DR^1^ for 28- or 14-days by area

	Ninohe PHC area			Yokote PHC area			Saku PHC area			Ishikawa PHC area			ANOVA P-value
				
	Mean	± SD	Median	Mean	± SD	Median	Mean	± SD	Median	Mean	± SD	Median	
Men	n=24			n=28			n=23			n=27			
SFA^2^	15.9	± 3.4	15.4	14.1	± 3.0	13.7	17.2	± 3.3	17.4	17.1	± 3.7	18.3	0.003
Myristic (14:0)	1.3	± 0.5	1.1	1.1	± 0.4	1.1	1.4	± 0.4	1.4	1.1	± 0.4	1.0	0.020
Palmitic (16:0)	9.8	± 1.7	10.1	8.7	± 1.6	8.7	10.4	± 1.8	10.4	10.7	± 2.2	11.1	0.001
Stearic (18:0)	3.5	± 0.7	3.5	3.1	± 0.6	3.1	3.7	± 0.8	3.7	4.2	± 1.0	4.3	0.000
MUFA^2^	19.0	± 3.5	19.1	17.7	± 2.9	16.9	20.2	± 3.4	20.3	21.4	± 4.3	22.1	0.001
Palmitoleic (16:1)	1.2	± 0.3	1.1	1.2	± 0.2	1.1	1.2	± 0.3	1.2	1.2	± 0.3	1.2	0.977
Oleic (18:1)	16.3	± 2.9	16.3	14.9	± 2.6	14.5	17.7	± 3.2	17.7	19.2	± 3.8	19.9	0.000
PUFA^2^	16.4	± 3.4	16.2	14.7	± 2.3	14.2	15.9	± 2.2	15.6	16.5	± 3.7	17.2	0.089
n-3 PUFA	3.9	± 1.3	3.8	3.5	± 0.7	3.4	3.6	± 0.6	3.7	2.9	± 0.8	2.8	0.002
Linolenic (18:3n-3)	1.9	± 0.4	1.9	1.7	± 0.3	1.7	1.8	± 0.3	1.8	2.0	± 0.5	2.0	0.140
EPA^2^ (20:5n-3)	0.7	± 0.4	0.7	0.6	± 0.2	0.6	0.6	± 0.2	0.6	0.3	± 0.2	0.3	0.000
DPA^2^ (22:5n-3)	0.2	± 0.1	0.1	0.2	± 0.0	0.1	0.1	± 0.0	0.1	0.1	± 0.0	0.1	0.000
DHA^2^ (22:6n-3)	1.0	± 0.5	1.0	1.0	± 0.3	1.0	1.0	± 0.3	1.0	0.6	± 0.3	0.5	0.000
n-6 PUFA	12.5	± 2.5	12.4	11.1	± 1.8	10.8	12.2	± 2.0	12.2	13.5	± 3	14.2	0.004
Linoleic (18:2n-6)	12.4	± 2.4	12.3	11.0	± 1.8	10.7	12.1	± 2.0	12.0	13.4	± 3	14.0	0.003
Eicosatrienoic (20:3n-6)	0.03	± 0.01	0.03	0.02	± 0.00	0.02	0.03	± 0.01	0.03	0.02	± 0.01	0.02	0.000
Arachidonic (20:4n-6)	0.2	± 0.0	0.2	0.2	± 0.0	0.2	0.2	± 0.0	0.2	0.2	± 0.0	0.2	0.025
n-6/n-3 ratio	3.4	± 0.7	3.4	3.2	± 0.6	3.1	3.5	± 0.8	3.5	4.7	± 0.8	4.8	0.000
PUFA/SFA ratio	1.1	± 0.2	1.1	1.1	± 0.2	1.1	0.9	± 0.1	0.9	1.0	± 0.2	1.0	0.125
TFA^2^	51.8	± 9.0	53.5	46.9	± 7.5	45.6	53.9	± 8.3	54.2	55.5	± 10.3	56.8	0.004

Women	n=27			n=30			n=28			n=28			
SFA	13.9	± 3.1	13.4	13.9	± 2.3	14.3	15.5	± 3.0	15.5	16.3	± 4.9	15.6	0.019
Myristic (14:0)	1.2	± 0.4	1.1	1.2	± 0.3	1.2	1.4	± 0.3	1.3	1.2	± 0.5	1.2	0.168
Palmitic (16:0)	8.3	± 1.5	8.2	8.4	± 1.3	8.5	9.1	± 1.6	9.2	9.7	± 2.8	9.4	0.018
Stearic (18:0)	3.1	± 0.7	3.1	3.0	± 0.6	3.1	3.4	± 0.7	3.3	3.9	± 1.3	3.8	0.001
MUFA	16.4	± 2.9	16.4	17.0	± 2.8	16.8	17.5	± 2.6	17.5	18.9	± 5.1	19.0	0.054
Palmitoleic (16:1)	1.0	± 0.2	0.9	1.1	± 0.2	1.1	1.0	± 0.2	1.0	1.0	± 0.4	1.0	0.322
Oleic (18:1)	14.2	± 2.7	14.3	14.5	± 2.5	14.3	15.4	± 2.3	15.5	17.0	± 4.5	17.2	0.004
PUFA	14.0	± 2.7	13.9	13.8	± 2.2	13.6	13.6	± 2.0	13.1	14.0	± 3.4	13.6	0.935
n-3 PUFA	3.2	± 0.8	3.0	3.2	± 0.6	3.1	3.0	± 0.6	3.0	2.5	± 0.7	2.3	0.000
Linolenic (18:3n-3)	1.6	± 0.4	1.7	1.6	± 0.3	1.6	1.7	± 0.3	1.7	1.7	± 0.4	1.7	0.961
EPA (20:5n-3)	0.6	± 0.2	0.5	0.6	± 0.2	0.5	0.4	± 0.1	0.4	0.3	± 0.1	0.2	0.000
DPA (22:5n-3)	0.1	± 0.0	0.1	0.1	± 0.0	0.1	0.1	± 0.0	0.1	0.1	± 0.0	0.1	0.000
DHA (22:6n-3)	0.8	± 0.3	0.7	0.8	± 0.2	0.8	0.7	± 0.2	0.8	0.5	± 0.2	0.4	0.000
n-6 PUFA	10.8	± 2.1	10.7	10.5	± 1.8	10.5	10.6	± 1.6	10.3	11.5	± 2.8	11.2	0.300
Linoleic (18:2n-6)	10.7	± 2.1	10.6	10.4	± 1.8	10.4	10.5	± 1.6	10.2	11.4	± 2.8	11.1	0.248
Eicosatrienoic (20:3n-6)	0.02	± 0.00	0.02	0.02	± 0.00	0.02	0.03	± 0.01	0.02	0.02	± 0.01	0.02	0.197
Arachidonic (20:4n-6)	0.2	± 0.0	0.2	0.2	± 0.0	0.2	0.2	± 0.0	0.2	0.1	± 0.0	0.1	0.435
n-6/n-3 ratio	3.4	± 0.7	3.6	3.3	± 0.5	3.2	3.6	± 0.5	3.5	4.8	± 0.8	4.9	0.000
PUFA/SFA ratio	1.0	± 0.2	1.0	1.0	± 0.1	1.0	0.9	± 0.2	0.9	0.9	± 0.2	0.9	0.019
TFA	44.7	± 7.6	45.7	45.1	± 6.9	44.9	47.1	± 6.8	47.4	49.6	± 12.6	48.5	0.145

^1^DR, dietary records.

^2^TFA, total fatty acid; SFA, saturated fatty acid; MUFA, monounsaturated fatty acid; PUFA, polyunsaturated fatty acid; EPA, eicosapentaenoic acid; DPA, docosapentaenoic acid; DHA, docosahexaenoic acid.

Table 2 shows the intake levels of fatty acid by sex and area estimated from FFQ. No apparent area-difference was observed in men or women for total fatty acid, SFA, and MUFA. Both in men and women, marine-origin n-3 fatty acid intake was lowest and the ratio of n-3 fatty acid intake to n-6 fatty acid intake was highest in Okinawa similar to the intake indicated by DR.

**Table 2.** Fatty acid intakes (g/day) assessed with FFQ^1^ by area

	Ninohe PHC area			Yokote PHC area			Saku PHC area			Ishikawa PHC area			ANOVA P-value
				
	Mean	± SD	Median	Mean	± SD	Median	Mean	± SD	Median	Mean	± SD	Median	
Men	n=24			n=28			n=23			n=27			
SFA^2^	21.7	± 13.2	17.4	15.5	± 7.5	13.9	17.7	± 6.2	17.4	16.7	± 6.9	17.6	0.076
14:0	1.9	± 1.7	1.5	1.2	± 0.7	1.0	1.5	± 0.7	1.4	1.2	± 0.6	1.1	0.070
16:0	11.6	± 6.6	9.5	8.3	± 3.9	7.3	9.4	± 3.4	9.1	9.3	± 3.9	9.5	0.082
18:0	4.9	± 2.8	4.0	3.5	± 1.9	2.9	3.9	± 1.5	4.0	4.0	± 1.9	4.2	0.121
MUFA^2^	27.5	± 14.3	24.2	20.9	± 9.3	18.6	22.8	± 8.1	23.9	22.6	± 10.0	24.8	0.161
16:1	1.4	± 0.9	1.1	1.0	± 0.4	0.9	1.0	± 0.4	1.1	1.1	± 0.5	1.1	0.054
18:1	23.1	± 12.1	20.7	17.8	± 8.3	15.6	19.6	± 7.3	20.7	19.8	± 8.9	21.6	0.236
PUFA^2^	18.1	± 8.4	15.8	14.2	± 6.3	12.9	14.7	± 4.7	15.9	11.4	± 4.9	11.3	0.004
n-3 PUFA	5.0	± 3.0	4.2	3.6	± 1.7	3.2	3.6	± 1.2	3.9	2.5	± 1.1	2.6	0.000
18:3n-3	2.5	± 1.3	2.3	2.0	± 1.0	1.9	2.1	± 0.8	2.2	1.8	± 0.9	1.7	0.089
20:5n-3	0.7	± 0.6	0.7	0.4	± 0.3	0.4	0.4	± 0.2	0.5	0.2	± 0.1	0.2	0.000
22:5n-3	0.2	± 0.1	0.2	0.1	± 0.1	0.1	0.1	± 0.0	0.1	0.1	± 0.0	0.1	0.000
22:6n-3	1.2	± 0.9	1.1	0.7	± 0.4	0.6	0.7	± 0.3	0.8	0.4	± 0.2	0.4	0.000
n-6 PUFA	13.1	± 5.6	11.9	10.5	± 4.7	9.3	11.1	± 3.6	12.0	8.9	± 3.8	8.4	0.014
18:2n-6	11.2	± 5.1	9.9	8.9	± 4.6	7.8	9.5	± 3.4	10.1	7.7	± 3.5	7.7	0.034
20:3n-6	0.03	± 0.02	0.03	0.02	± 0.01	0.02	0.02	± 0.01	0.02	0.02	± 0.01	0.02	0.002
20:4n-6	0.2	± 0.1	0.2	0.1	± 0.1	0.1	0.1	± 0.1	0.1	0.1	± 0.1	0.1	0.001
n-6/n-3 ratio	2.9	± 0.7	2.7	3.0	± 0.6	2.9	3.1	± 0.6	3.1	3.6	± 0.7	3.5	0.001
PUFA/SFA ratio	0.9	± 0.3	0.9	1.0	± 0.2	0.9	0.9	± 0.3	0.9	0.7	± 0.1	0.7	0.001
TFA^2^	67.4	± 33.4	58.6	50.7	± 22.4	44.9	55.2	± 18.0	55.3	50.8	± 21.5	53.8	0.057

Women	n=27			n=30			n=28			n=28			
SFA	18.6	± 8.4	17.5	15.3	± 5.6	14.3	20.3	± 13.6	16.4	16.4	± 6.8	14.2	0.150
14:0	1.6	± 0.8	1.5	1.2	± 0.6	1.0	1.7	± 1.3	1.3	1.5	± 0.7	1.3	0.159
16:0	9.9	± 4.7	8.8	8.2	± 3.0	7.6	10.9	± 7.6	8.7	8.6	± 3.5	7.6	0.150
18:0	4.2	± 2.1	3.5	3.4	± 1.4	3.1	4.5	± 3.1	3.6	3.7	± 1.7	3.2	0.203
MUFA	24.7	± 13.1	19.9	20.3	± 7.7	18.8	27.5	± 22.0	22.6	19.9	± 8.0	19.2	0.127
16:1	1.1	± 0.6	1.0	0.9	± 0.4	0.9	1.2	± 1.0	1.0	0.9	± 0.4	0.8	0.142
18:1	21.0	± 11.4	16.6	17.4	± 6.8	16.0	23.9	± 19.1	19.6	17.5	± 7.1	16.8	0.139
PUFA	16.7	± 8.6	13.1	13.7	± 5.6	12.3	17.7	± 17.3	13.4	10.6	± 3.5	10.4	0.043
n-3 PUFA	4.6	± 2.9	3.7	3.5	± 1.7	3.3	4.6	± 5.0	3.3	2.4	± 0.9	2.2	0.019
18:3n-3	2.5	± 1.5	1.8	2.1	± 1.0	1.9	2.6	± 2.5	2.0	1.7	± 0.6	1.6	0.122
20:5n-3	0.6	± 0.5	0.4	0.4	± 0.3	0.3	0.6	± 0.9	0.4	0.2	± 0.1	0.1	0.003
22:5n-3	0.2	± 0.1	0.1	0.1	± 0.1	0.1	0.1	± 0.2	0.1	0.0	± 0.0	0.0	0.005
22:6n-3	1.0	± 0.8	0.7	0.7	± 0.4	0.6	0.9	± 1.2	0.7	0.3	± 0.2	0.3	0.003
n-6 PUFA	12.1	± 5.9	10.1	10.2	± 4.0	9.3	13.1	± 12.3	10.0	8.2	± 2.7	8.4	0.062
18:2n-6	10.7	± 5.6	8.7	8.7	± 3.8	7.9	11.7	± 12.0	9.0	7.2	± 2.5	7.3	0.079
20:3n-6	0.02	± 0.01	0.02	0.02	± 0.01	0.02	0.03	± 0.02	0.02	0.02	± 0.01	0.02	0.053
20:4n-6	0.2	± 0.1	0.1	0.1	± 0.1	0.1	0.2	± 0.2	0.2	0.1	± 0.0	0.1	0.015
n-6/n-3 ratio	2.9	± 0.7	2.8	3.0	± 0.5	3.1	3.0	± 0.5	3.0	3.6	± 0.7	3.3	0.000
PUFA/SFA ratio	0.9	± 0.3	0.8	0.9	± 0.2	0.9	0.9	± 0.3	0.8	0.7	± 0.1	0.7	0.000
TFA	60.1	± 28.9	50.7	49.3	± 18.1	45.6	65.6	± 52	52.1	47.0	± 17.9	43.6	0.101

^1^FFQ, food frequency questionnaire.

^2^TFA, total fatty acid; SFA, saturated fatty acid; MUFA, monounsaturated fatty acid; PUFA, polyunsaturated fatty acid.

Table 3 shows the mean and median fatty acid composition of serum phospholipid by area. SFA and MUFA compositions were lowest and that of PUFA was highest in Iwate. Marine-origin n-3 fatty acid intake was lowest and the ratio of n-3 fatty acid intake to n-6 fatty acid intake was highest in Okinawa similar to the intake according to both DR and FFQ.

**Table 3.** Serum phospholipid fatty acid level (% of TFA^1^) in men by area

	Ninohe PHC area			Yokote PHC area			Saku PHC area			Ishikawa PHC area			ANOVA P-value
				
	Mean	± SD	Median	Mean	± SD	Median	Mean	± SD	Median	Mean	± SD	Median	
	n=22			n=25			n=19			n=22			
SFA^1^	46.3	± 3.6	45.9	48.8	± 2.5	49.1	46.9	± 2.4	46.9	47.3	± 1.6	47.0	0.015
14:0	0.4	± 0.1	0.4	0.4	± 0.1	0.4	0.5	± 0.3	0.5	0.3	± 0.2	0.4	0.082
16:0	28.2	± 2.4	28.6	29.8	± 2.4	29.5	28.7	± 1.5	28.8	28.0	± 2.5	27.3	0.029
18:0	14.8	± 2.0	14.4	15.3	± 1.0	15.3	14.6	± 2.0	14.1	15.6	± 1.5	15.7	0.181
MUFA^1^	12.7	± 1.8	12.1	13.8	± 1.1	13.8	13.6	± 1.4	13.2	14.2	± 1.8	13.7	0.015
16:1	0.6	± 0.3	0.5	0.6	± 0.2	0.6	0.5	± 0.2	0.5	0.7	± 0.4	0.6	0.489
18:1	8.1	± 1.3	7.7	8.8	± 1.0	8.6	9.1	± 1.1	8.9	9.2	± 1.5	8.8	0.013
PUFA^1^	40.9	± 4.2	42.4	37.2	± 2.7	37.4	39.5	± 3.2	40.5	38.4	± 2.2	39.0	0.001
n-3 PUFA	14.4	± 3.2	14.5	13.4	± 2.8	13.8	12.5	± 2.7	12.4	9.0	± 1.4	8.7	0.000
18:3n-3	0.3	± 0.1	0.2	0.2	± 0.2	0.2	0.2	± 0.1	0.3	0.2	± 0.2	0.2	0.950
20:5n-3	4.5	± 1.5	4.6	4.5	± 1.5	4.4	3.3	± 1.1	3.2	2.1	± 0.8	2.0	0.000
22:5n-3	1.5	± 0.3	1.5	1.2	± 0.3	1.1	1.3	± 0.3	1.4	0.8	± 0.1	0.8	0.000
22:6n-3	8.1	± 1.8	8.2	7.5	± 1.5	7.4	7.6	± 1.8	7.7	5.9	± 0.8	5.8	0.000
n-6 PUFA	26.5	± 3.0	26.7	23.8	± 3.8	23.7	27.0	± 2.6	27.4	29.3	± 2.3	29.3	0.000
18:2n-6	17.8	± 2.9	17.8	15.6	± 3.4	14.9	18.0	± 2.8	18.5	18.4	± 2.4	18.8	0.005
20:3n-6	1.7	± 0.3	1.7	1.5	± 0.3	1.5	1.7	± 0.5	1.8	2.1	± 0.5	2.0	0.000
20:4n-6	5.8	± 1.3	5.5	6.0	± 1.0	6.0	6.0	± 1.1	5.9	7.9	± 1.2	7.8	0.000
n-6/n-3 ratio	2.0	± 0.5	2.0	1.9	± 0.7	1.7	2.3	± 0.6	2.2	3.4	± 0.7	3.4	0.000
n-3/20:4n-6 ratio	2.6	± 0.6	2.5	2.4	± 0.7	2.2	2.2	± 0.6	1.9	1.1	± 0.3	1.1	0.000
PUFA/SFA ratio	0.9	± 0.1	0.9	0.8	± 0.1	0.8	0.8	± 0.1	0.9	0.8	± 0.1	0.8	0.001

^1^TFA, total fatty acid; SFA, saturated fatty acid; MUFA, monounsaturated fatty acid;. PUFA, polyunsaturated fatty acid.

Table 4 shows fatty acid intake levels estimated from DR and FFQ in 4 areas. Both in men and women, SFA and MUFA intakes estimated from FFQ were higher than intake from DR. Marine-origin n-3 fatty acid intake estimated from FFQ was lower than intake from DR in men, although no apparent difference was observed in women. Table 4 also presents the relations between fatty acid intake estimated from FFQ and fatty acid intake estimated from DR. For comparison, intake of fatty acid was expressed in three ways: crude intake (g/day), energy-adjusted value, and percentage of total fatty acid intake. Both in men and women, SFA and MUFA intakes estimated from FFQ were highly correlated with intake from DR when intake was expressed as an energy-adjusted value (r=0.61 and 0.60 for SFA, r=0.50 and r=0.44 for MUFA in men and women, respectively). Of the PUFA intake, marine-origin n-3 fatty acid intake estimated from FFQ was well correlated with intake from DR expressed in all three ways, and was especially higher when intake was expressed as a percentage of total fatty acid (r=0.62 and 0.55 for EPA, r=0.61 and r=0.50 for DHA in men and women, respectively).

**Table 4.** Fatty acid intakes (g/day) assessed with DR^3^ for 28- or 14-days and FFQ^4^ in 4 areas and correlations

	DR			FFQ			% difference	Spearman correlation		
			
	Mean	± SD	Median	Mean	± SD	Median		Crude	Energy-adjusted^1^	% of TFA^2^
Men (n=102)										
SFA^2^	16.0	± 3.5	16.2	17.8	± 9.0	16.6	11	0.43	0.61	0.43
14:0	1.2	± 0.4	1.2	1.4	± 1.0	1.2	19	0.52	0.53	0.48
16:0	9.9	± 2.0	10.1	9.6	± 4.7	9.0	-3	0.38	0.61	0.30
18:0	3.6	± 0.9	3.7	4.0	± 2.1	3.7	11	0.44	0.63	0.53
MUFA^2^	19.6	± 3.8	19.4	23.3	± 10.8	22.3	19	0.30	0.50	0.30
16:1	1.2	± 0.3	1.1	1.1	± 0.6	1.0	-5	0.20	0.32	0.11
18:1	17.0	± 3.5	17.0	20.0	± 9.4	19.2	18	0.33	0.53	0.53
PUFA^2^	15.8	± 3.0	15.6	14.5	± 6.6	13.2	-9	0.16	0.27	0.43
n-3 PUFA	3.5	± 0.9	3.4	3.7	± 2.1	3.3	6	0.27	0.21	0.65
18:3n-3	1.8	± 0.4	1.8	2.1	± 1.0	1.9	12	0.13	0.27	0.11
20:5n-3	0.6	± 0.3	0.5	0.4	± 0.4	0.3	-20	0.44	0.38	0.62
22:5n-3	0.1	± 0.1	0.1	0.1	± 0.1	0.1	-15	0.38	0.32	0.59
22:6n-3	0.9	± 0.4	0.8	0.7	± 0.6	0.5	-15	0.40	0.34	0.61
n-6 PUFA	12.3	± 2.5	12.1	10.8	± 4.7	10.0	-12	0.15	0.30	0.21
18:2n-6	12.2	± 2.5	12.0	9.2	± 4.3	8.3	-24	0.16	0.29	0.12
20:3n-6	0.0	± 0.0	0.0	0.0	± 0.0	0.0	-11	0.42	0.39	0.29
20:4n-6	0.2	± 0.0	0.2	0.2	± 0.1	0.1	-17	0.28	0.32	0.32
n-6/n-3 ratio	3.7	± 0.9	3.6	3.2	± 0.7	3.2	-14	0.40		
PUFA/SFA ratio	1.0	± 0.2	1.0	0.9	± 0.3	0.8	-16	0.41		
TFA^2^	51.9	± 9.3	53.2	55.7	± 25.0	52.8	7	0.29	0.52	

Women (n=113)										
SFA	14.9	± 3.5	14.6	17.6	± 9.2	15.3	18	0.26	0.60	0.45
14:0	1.2	± 0.4	1.2	1.5	± 0.9	1.3	22	0.44	0.53	0.38
16:0	8.9	± 1.9	8.6	9.4	± 5.0	8.1	5	0.22	0.59	0.43
18:0	3.4	± 0.9	3.2	3.9	± 2.1	3.4	17	0.21	0.58	0.46
MUFA	17.5	± 3.6	17.0	23.0	± 14.1	19.3	32	0.13	0.44	0.30
16:1	1.0	± 0.3	1.0	1.0	± 0.7	0.9	3	0.21	0.34	0.28
18:1	15.2	± 3.3	15.0	19.9	± 12.2	16.8	31	0.14	0.48	0.40
PUFA	13.8	± 2.6	13.5	14.6	± 10.4	12.2	6	0.16	0.24	0.50
n-3 PUFA	3.0	± 0.7	2.9	3.7	± 3.1	2.9	26	0.31	0.34	0.56
18:3n-3	1.7	± 0.4	1.7	2.2	± 1.6	1.8	33	0.08	0.25	0.19
20:5n-3	0.5	± 0.2	0.4	0.4	± 0.6	0.3	-2	0.45	0.45	0.55
22:5n-3	0.1	± 0	0.1	0.1	± 0.1	0.1	4	0.40	0.39	0.51
22:6n-3	0.7	± 0.3	0.7	0.7	± 0.8	0.5	2	0.41	0.37	0.50
n-6PUFA	10.8	± 2.1	10.7	10.9	± 7.4	9.4	1	0.11	0.21	0.34
18:2n-6	10.7	± 2.1	10.6	9.6	± 7.1	8.1	-11	0.10	0.22	0.27
20:3n-6	0.0	± 0.0	0.0	0.0	± 0.0	0.0	-4	0.40	0.43	0.27
20:4n-6	0.2	± 0.0	0.2	0.2	± 0.1	0.1	-6	0.24	0.25	0.29
n-6/n-3 ratio	3.8	± 0.8	3.6	3.2	± 0.7	3.1	-16	0.37		
PUFA/SFA ratio	1.0	± 0.2	0.9	0.8	± 0.2	0.8	-13	0.49		
TFA	46.6	± 8.9	46.2	55.4	± 32.6	48.3	19	0.16	0.47	

^1^Energy was adjusted by residual model for intake.

^2^TFA, total fatty acid; SFA, saturated fatty acid; MUFA, monounsaturated fatty acid; PUFA, polyunsaturated fatty acid.

^3^DR, dietary records.

^4^FFQ, food frequency questionnaire.

For n=102, r>0.20 = p<0.05, r>0.26 = p<0.01, r>0.33 = p<0.001.

For n=113, r>0.19 = p<0.05, r>0.25 = p<0.01, r>0.31 = p<0.001.

Table 5 shows the mean fatty acid intake estimated from DR within quintiles of corresponding fatty acid intake estimated from FFQ. Both in men and women, SFA and marine-origin n-3 fatty acid intake from DR in the highest quintile was significantly higher than the corresponding fatty acid intake in the lowest quintiles. The result of cross-classification of the subjects by quintiles from the fatty acid intake from DR and fatty acid intake from FFQ are shown in Table 6. As for all fatty acid, more than 50% of the subjects were classified in the adjacent quintiles and less than 10% of them were classified in the extreme quintiles.

**Table 5.** Fatty acid intakes assessed with DR^2^ classified with quintiles of the intakes assessed with FFQ^2^ for corresponding fatty acids (g/day)

	Lowest		Second			Third			Fourth			Highest		
					
	mean	± SD	mean	± SD	ratio^4^	mean	± SD	ratio^4^	mean	± SD	ratio^4^	mean	± SD	ratio^4^
Men	n=20		n=21			n=20			n=21			n=20		
SFA^3^	14.0	± 3.6	14.7	± 2.6	1.05	16.3	± 3.2	1.16	16.7	± 3.2	1.19	18.4	± 3.6	1.31
14:0	1.0	± 0.3	1.0	± 0.2	0.95	1.2	± 0.4	1.19	1.3	± 0.3	1.26	1.6	± 0.5	1.62
16:0	8.9	± 2.3	9.7	± 1.6	1.09	9.8	± 1.7	1.11	10.0	± 1.9	1.13	11.1	± 1.8	1.25
18:0	3.2	± 0.9	3.5	± 0.8	1.11	3.5	± 0.7	1.11	3.7	± 0.8	1.17	4.3	± 0.8	1.36
MUFA^3^	18.2	± 4.6	19.1	± 3.9	1.05	18.4	± 2.8	1.01	21.2	± 2.8	1.17	20.8	± 3.8	1.15
16:1	1.1	± 0.3	1.2	± 0.2	1.16	1.1	± 0.2	1.08	1.1	± 0.2	1.03	1.3	± 0.3	1.26
18:1	16.0	± 4.6	15.7	± 3.0	0.98	16.4	± 3.0	1.03	18.6	± 2.8	1.17	18.2	± 3.3	1.14
PUFA^3^	15.3	± 4.4	15.9	± 2.7	1.04	15.5	± 2.4	1.02	15.9	± 2.2	1.04	16.6	± 3.2	1.08
n-3 PUFA	3.1	± 0.9	3.3	± 0.6	1.06	3.4	± 0.8	1.10	3.5	± 0.6	1.12	4.0	± 1.3	1.26
18:3n-3	1.8	± 0.6	1.8	± 0.3	1.04	1.9	± 0.4	1.06	1.8	± 0.3	1.04	1.9	± 0.4	1.08
20:5n-3	0.4	± 0.2	0.5	± 0.2	1.13	0.5	± 0.2	1.34	0.6	± 0.2	1.50	0.7	± 0.4	1.78
22:5n-3	0.1	± 0.0	0.1	± 0.1	1.17	0.1	± 0.1	1.32	0.1	± 0.0	1.34	0.2	± 0.1	1.72
22:6n-3	0.7	± 0.3	0.7	± 0.3	1.02	0.9	± 0.3	1.25	0.9	± 0.2	1.26	1.2	± 0.5	1.61
n-6 PUFA	11.8	± 3.7	12.4	± 2.4	1.05	12.4	± 2.3	1.05	12.2	± 1.7	1.03	12.8	± 2.2	1.09
18:2n-6	11.3	± 3.4	12.6	± 2.6	1.11	12.3	± 2.4	1.09	12.1	± 1.6	1.07	12.7	± 2.2	1.12
20:3n-6	0.0	± 0.0	0.0	± 0.0	1.00	0.0	± 0.0	1.16	0.0	± 0.0	1.14	0.0	± 0.0	1.34
20:4n-6	0.2	± 0.0	0.2	± 0.0	1.04	0.2	± 0.0	1.03	0.2	± 0.0	1.14	0.2	± 0.0	1.23
n-6/n-3 ratio	3.1	± 0.7	3.7	± 1.1	1.21	3.8	± 1.0	1.25	3.7	± 0.6	1.20	4.2	± 0.8	1.39
n-3/20:4n-6 ratio	16.5	± 2.8	17.8	± 2.8	1.08	19.9	± 5.5	1.20	19.0	± 3.1	1.15	19.7	± 2.9	1.19
PUFA/SFA ratio	0.9	± 0.2	0.9	± 0.2	1.05	1.0	± 0.2	1.15	1.0	± 0.2	1.16	1.2	± 0.2	1.29
TFA^3^	49.2	± 12.7	49.8	± 7.6	1.01	50.5	± 7.7	1.03	55.2	± 7.0	1.12	54.6	± 9.9	1.11

Women	n=22		n=23			n=23			n=23			n=22		
SFA	13.3	± 2.8	15.7	± 4.6	1.18	14.5	± 2.1	1.09	14.7	± 3.9	1.11	16.3	± 3.2	1.23
14:0	1.0	± 0.3	1.1	± 0.3	1.13	1.2	± 0.5	1.23	1.3	± 0.3	1.27	1.5	± 0.4	1.48
16:0	8.2	± 1.8	9.2	± 2.3	1.12	8.7	± 1.5	1.06	8.9	± 2.1	1.09	9.5	± 1.7	1.17
18:0	3.0	± 0.8	3.4	± 1.1	1.12	3.4	± 0.7	1.10	3.3	± 0.9	1.08	3.6	± 0.8	1.20
MUFA	16.8	± 3.9	17.8	± 4.2	1.06	17.1	± 2.4	1.02	17.3	± 3.6	1.03	18.4	± 3.5	1.10
16:1	0.9	± 0.3	1.0	± 0.4	1.13	1.0	± 0.2	1.08	1.0	± 0.2	1.09	1.1	± 0.3	1.14
18:1	14.3	± 3.6	15.8	± 3.7	1.11	14.7	± 2.2	1.03	15.3	± 3.3	1.07	16.0	± 3.4	1.12
PUFA	13.4	± 2.7	13.2	± 2.6	0.99	14.0	± 2.9	1.05	14.4	± 2.8	1.07	14.1	± 2.0	1.05
n-3 PUFA	2.7	± 0.7	2.8	± 0.7	1.05	3.0	± 0.6	1.15	3.0	± 0.7	1.15	3.4	± 0.8	1.26
18:3n-3	1.6	± 0.4	1.6	± 0.4	0.99	1.6	± 0.3	1.00	1.7	± 0.3	1.02	1.7	± 0.4	1.06
20:5n-3	0.3	± 0.2	0.4	± 0.2	1.36	0.5	± 0.2	1.76	0.5	± 0.2	1.75	0.5	± 0.2	1.87
22:5n-3	0.1	± 0.0	0.1	± 0.0	1.41	0.1	± 0.0	1.67	0.1	± 0.0	1.69	0.1	± 0.0	1.76
22:6n-3	0.5	± 0.2	0.6	± 0.2	1.32	0.8	± 0.2	1.67	0.8	± 0.2	1.57	0.8	± 0.3	1.71
n-6 PUFA	10.4	± 2.3	10.6	± 2.1	1.02	10.5	± 2.1	1.01	11.7	± 2.4	1.12	10.8	± 1.7	1.03
18:2n-6	10.6	± 2.2	10.5	± 2.5	0.99	10.8	± 1.9	1.02	11.0	± 2.4	1.04	10.8	± 1.7	1.02
20:3n-6	0.0	± 0.0	0.0	± 0.0	1.11	0.0	± 0.0	1.14	0.0	± 0.0	1.19	0.0	± 0.0	1.32
20:4n-6	0.2	± 0.0	0.1	± 0.0	0.89	0.2	± 0.0	1.00	0.2	± 0.0	1.14	0.2	± 0.0	1.05
n-6/n-3 ratio	3.3	± 0.7	3.7	± 0.7	1.11	3.7	± 0.9	1.12	3.5	± 0.6	1.06	4.5	± 0.9	1.36
n-3/20:4n-6 ratio	17.6	± 4.3	17.9	± 2.4	1.02	18.6	± 3.5	1.05	20.3	± 3.4	1.15	20.5	± 4.1	1.17
PUFA/SFA ratio	0.9	± 0.1	0.8	± 0.2	0.98	1.0	± 0.2	1.16	1.0	± 0.2	1.22	1.1	± 0.2	1.26
TFA	44.2	± 9.6	47.8	± 9.7	1.08	43.4	± 7.2	0.98	49.6	± 9.5	1.12	48.2	± 7.1	1.09

^1^DR, dietary records.

^2^FFQ, food frequency questionnaire.

^3^TFA, total fatty acid; SFA, saturated fatty acid; MUFA, monounsaturated fatty acid; PUFA, polyunsaturated fatty acid.

^4^Ratio compared to the lowest quintile.

**Table 6.** Comparison of FFQ^1^ with DR^2^ for fatty acid intake based on joint classification by quintiles (%)

	Men (n=102)			Women (n=113)		
		
	Same category	Adjacent category	Extreme category	Same category	Adjacent category	Extreme category
SFA^3^	38	69	3	29	65	2
14:0	36	70	0	34	67	1
16:0	39	69	3	30	63	3
18:0	33	70	3	30	63	5
MUFA^3^	28	71	8	35	61	6
16:1	28	57	5	32	61	7
18:1	29	69	9	28	59	6
PUFA^3^	29	59	6	20	58	5
n-3 PUPA	31	65	5	26	62	4
18:3n-3	25	58	6	20	58	6
20:5n-3	28	70	2	30	68	1
22:5n-3	30	65	3	27	62	2
22:6n-3	29	68	3	28	64	1
n-6 PUPA	21	59	7	16	58	6
18:2n-6	22	61	6	22	55	6
20:3n-6	39	67	3	32	65	1
20:4n-6	24	56	4	30	63	4
n-6/n-3 ratio	28	63	1	31	64	1
n-3/20:4n-6 ratio	29	67	4	30	68	3
PUFA/SFA ratio	30	66	2	31	67	1
TFA^3^	30	67	8	27	63	6

^1^FFQ, food frequency questionnaire.

^2^DR, dietary records.

^3^TFA, total fatty acid; SFA, saturated fatty acid; MUFA, monounsaturated fatty acid; PUFA, polyunsaturated fatty acid.

Table 7 shows the mean and median fatty acid composition of serum phospholipid in 4 areas and the relation between the serum phospholipid fatty acid level and fatty acid intake estimated from both FFQ and DR. The correlations of SFA composition in serum phospholipid with the respective intake estimated both from FFQ and DR were weak, although for MUFA, a significant correlation was observed for DR (r=0.39), when intake was expressed as a percentage of total FA intake. Marine-origin n-3 fatty acid compositions in serum phospholipid were well-correlated with the respective intake both estimated from FFQ and DR, and especially higher when intake was estimated from DR. When estimated with FFQ and DR, the correlations were higher when intake was expressed as a percentage of total FA intake (r=0.59 and 0.76 for EPA, r=0.49 and r=0.50 for DHA in FFQ and DR, respectively).

**Table 7.** Serum phospholipid fatty acid level (% of TFA) in 4 areas and correlations with fatty acid intakes assessed with either DR^3^ or FFQ^4^ in men (n=88)

				Spearman correlation					
	
				DR			FFQ		
		
	Mean	± SD	Median	crude	energy-adjusted^1^	% of TFA^2^	crude	energy-adjusted^1^	% of TFA^2^
SFA^2^	47.4	± 2.8	47.0	-0.01	0.02	-0.08	-0.15	-0.09	-0.01
14:0	0.4	± 0.2	0.4	0.02	0.08	0.06	0.05	0.00	0.08
16:0	28.7	± 2.3	28.8	-0.20	-0.28	-0.02	-0.32	-0.44	-0.21
18:0	15.1	± 1.7	15.0	0.07	0.13	-0.01	0.09	0.23	0.11
MUFA^2^	13.6	± 1.6	13.4	0.00	0.08	0.39	-0.13	0.04	0.19
16:1	0.6	± 0.3	0.6	0.05	0.01	0.22	-0.18	-0.16	0.10
18:1	8.8	± 1.3	8.5	-0.02	0.04	0.36	-0.15	-0.05	0.19
PUFA^2^	38.9	± 3.4	39.3	0.14	0.02	0.09	0.29	0.19	0.16
n-3 PUFA	12.4	± 3.3	12.4	0.46	0.31	0.69	0.29	0.21	0.64
18:3n-3	0.2	± 0.2	0.2	0.03	-0.05	0.14	-0.14	-0.09	-0.03
20:5n-3	3.7	± 1.6	3.4	0.75	0.73	0.76	0.43	0.44	0.59
22:5n-3	1.2	± 0.4	1.2	0.47	0.33	0.51	0.45	0.39	0.56
22:6n-3	7.3	± 1.7	7.1	0.43	0.42	0.50	0.35	0.32	0.49
n-6 PUFA	26.5	± 3.6	26.9	0.25	0.44	0.03	0.04	0.20	-0.37
18:2n-6	17.4	± 3.1	17.6	0.20	0.27	-0.01	0.16	0.18	-0.12
20:3n-6	1.7	± 0.5	1.7	-0.18	-0.14	-0.34	0.00	0.05	-0.23
20:4n-6	6.4	± 1.4	6.2	-0.20	0.18	-0.22	-0.13	0.00	-0.22
n-6/n-3 ratio	2.4	± 0.9	2.2	0.71			0.45		
n-3/20:4n-6 ratio	2.1	± 0.8	2.1	0.41			0.41		
PUFA/SFA ratio	0.8	± 0.1	0.8	-0.02			0.08		

^1^Energy was adjusted by residual model for intake.

^2^TFA, total fatty acid; SFA, saturated fatty acid; MUFA, monounsaturated fatty acid; PUFA, polyunsaturated fatty acid.

^3^DR, dietary records.

^4^FFQ, food frequency questionnaire.

For n=88, r>0.21 = p<0.05, r>0.28 = p<0.01, r>0.35 = p<0.001.

Table 8 shows the mean serum phospholipid fatty acid level (% of total fatty acid) within quintiles of corresponding fatty acid intake estimated from FFQ for men. Marine-origin n-3 fatty acid composition of serum phospholipid in the highest or next highest quintiles was significantly higher than the corresponding composition of serum phospholipid in the lowest quintiles. The results of cross-classification of the subjects by quintiles from the serum phospholipid fatty acid and fatty acid intake from FFQ are shown in Table 9. More than 70% of the subjects were classified in the adjacent quintiles in terms of the marine-origin n-3 fatty acids.

**Table 8.** Serum phospholipid fatty acid level classified with quintiles of the intakes assessed with FFQ^1^ for corresponding fatty acids (% of TFA^2^) in men

	Lowest		Second			Third			Fourth			Highest		
				
	mean	± SD	mean	± SD	ratio^3^	mean	± SD	ratio^3^	mean	± SD	ratio^3^	mean	± SD	ratio^3^
	n=17		n=18			n=18			n=18			n=17		
SFA^2^	47.0	± 2.7	48.0	± 2.1	1.02	47.8	± 3.3	1.02	47.1	± 3.3	1.00	47.1	± 2.2	1.00
14:0	0.4	± 0.1	0.4	± 0.1	1.20	0.4	± 0.1	1.05	0.4	± 0.2	1.10	0.5	± 0.4	1.33
16:0	29.3	± 2.4	28.9	± 2.1	0.99	28.8	± 1.7	0.98	28.3	± 2.8	0.97	28.2	± 2.6	0.96
18:0	15.0	± 1.7	14.6	± 1.6	0.98	15.4	± 2.1	1.03	15.2	± 1.5	1.01	15.4	± 1.4	1.03
MUFA^2^	12.7	± 1.3	13.9	± 1.7	1.09	13.6	± 1.9	1.07	13.8	± 1.1	1.09	13.9	± 1.6	1.09
16:1	0.6	± 0.3	0.6	± 0.3	1.09	0.5	± 0.2	0.85	0.7	± 0.3	1.18	0.6	± 0.2	0.99
18:1	8.1	± 0.9	8.8	± 1.3	1.09	9.1	± 1.3	1.12	8.8	± 1.3	1.09	9.1	± 1.4	1.12
PUFA^2^	38.6	± 2.8	38.5	± 3.6	1.00	38.6	± 3.6	1.00	38.5	± 3.8	1.00	40.2	± 3.0	1.04
n-3 PUFA	10.0	± 2.3	10.2	± 2.4	1.02	12.1	± 2.7	1.21	13.9	± 3.1	1.39	15.5	± 2.5	1.55
18:3n-3	0.3	± 0.2	0.2	± 0.1	0.89	0.2	± 0.1	0.81	0.2	± 0.1	0.80	0.3	± 0.3	1.07
20:5n-3	2.3	± 0.8	3.0	± 1.2	1.34	3.6	± 1.3	1.58	4.1	± 1.6	1.80	5.4	± 1.3	2.38
22:5n-3	0.8	± 0.2	1.1	± 0.4	1.26	1.2	± 0.4	1.40	1.4	± 0.3	1.68	1.4	± 0.3	1.66
22:6n-3	6.1	± 1.1	6.9	± 1.2	1.13	6.8	± 1.7	1.12	8.2	± 1.7	1.36	8.4	± 1.6	1.38
n-6 PUFA	28.4	± 2.5	27.9	± 3.6	0.98	25.7	± 3.8	0.91	25.8	± 2.9	0.91	24.8	± 4.0	0.87
18:2n-6	17.7	± 3.0	17.4	± 3.4	0.98	18.4	± 3.4	1.04	16.5	± 2.4	0.93	16.8	± 3.1	0.94
20:3n-6	1.8	± 0.5	1.9	± 0.5	1.08	1.8	± 0.5	0.98	1.5	± 0.4	0.82	1.7	± 0.5	0.92
20:4n-6	6.6	± 1.7	7.1	± 1.5	1.08	6.3	± 1.5	0.95	6.2	± 0.9	0.94	5.8	± 1.3	0.88

^1^FFQ, food frequency questionnaire.

^2^TFA, total fatty acid; SFA, saturated fatty acid; MUFA, monounsaturated fatty acid; PUFA, polyunsaturated fatty acid.

^3^Ratio compared to the lowest quintile.

**Table 9.** Comparison of FFQ^1^ for fatty acid intake with serum phospholipid fatty acid level based on joint classification by quintiles (%)

	Men (n=88)		
	
	Same category	Adjacent category	Extreme category
SFA^2^	24	52	8
14:0	16	50	2
16:0	15	43	13
18:0	35	69	7
MUFA^2^	24	61	5
16:1	26	51	9
18:1	25	61	2
PUFA^2^	28	58	5
n-3 PUFA	33	75	0
18:3n-3	25	55	9
20:5n-3	40	77	0
22:5n-3	32	78	0
22:6n-3	31	69	0
n-6 PUFA	10	35	14
18:2n-6	16	44	10
20:3n-6	17	41	10
20:4n-6	16	55	10

^1^FFQ, food frequency questionnaire.

^2^SFA, saturated fatty acid; MUFA, monounsaturated fatty acid; PUFA, polyunsaturated fatty acid.

Tables 10-14 show the cumulative percent contributions of the top 20 foods for SFA, MUFA, PUFA, n-3 fatty acid, and n-6 fatty acid, respectively. Dairy and meat products were important contributors of SFA. Vegetable oils were the largest contributor and meat was also an important contributor of MUFA. Although vegetable oils were the largest contributor, lean foods such as rice, miso, and tofu were also important contributors of PUFA and n-6 fatty acids. Even though vegetable oils were the largest contributors, many kinds of fish were also important contributors of n-3 fatty acid.﻿﻿

**Table 10.** Cumulative % contribution of the top 20 foods for saturated fatty acid assessed by DR

Food code^1^	Description^1^	g/day	Cumulative^2^ percent
Men (n=102)			
11-2	Liquid milk/Ordinary liquid milk	2.3	14.3
5-15	Vegetable oils/Vegetable oil, mixed	1.2	22.1
10-5a	Chicken egg/Whole egg/fresh	1.2	29.5
1-41d	Rice (Paddy rice)/Grains/Well-milled rice	0.9	35.2
9-70a	Pork/Belly without separable fat	0.8	40.3
11-28	Butter, salted	0.4	42.9
9-13b	Beef/Frank, Short plate, total edible	0.3	45.0
7-21a	Tofu, soybean curd/Momen	0.3	46.9
9-22	Beef and separable fat/Ground meat	0.3	48.6
11-23	Cheese/Process cheese	0.3	50.3
9-68a	Pork and separable fat/Loin, separable lean	0.2	51.8
8-84a	Mackerel/Raw/Uncooked	0.2	53.4
9-85a	Pork products/Bacon	0.2	54.8
9-47b	Chicken/Breast, total edible/Broiler	0.2	56.1
17-16b	Spices/Curry/Roux	0.2	57.4
9-49b	Chicken and separable fat/Thigh/Broiler	0.2	58.7
9-7b	Beef/Chuck loin, total edible	0.2	59.9
9-87f	Pork products/Sausage/Lyoner	0.2	61.2
1-31b	Precooked Chinese noodles/Dried by frying	0.2	62.3
9-66a	Pork/Boston butt, separable lean	0.2	63.4
Women (n=113)			
11-2	Liquid milk/Ordinary liquid milk	2.6	17.7
5-15	Vegetable oils/Vegetable oil, mixed	1.1	24.8
10-5a	Chicken egg/Whole egg/fresh	1.0	31.4
9-70a	Pork and separable fat/Belly without separable fat/Large-type breeds	0.8	36.5
1-41d	Rice (Paddy rice)/Grains/Well-milled rice: yield 90-92%	0.6	40.5
11-28	Other Milk and Dairy Products/Butter, salted	0.4	43.4
4-46	Western-style undried and semi-dried confectioneries/Shortcake	0.3	45.1
11-23	Cheese/Process cheese	0.2	46.7
7-21a	Tofu and Abura-age/Tofu, soybean curd/Momen	0.2	48.4
9-13b	Beef and separable fat/Frank, Short plate, total edible/Dairy-fattened steer	0.2	50.1
9-85a	Pork products/Bacon	0.2	51.5
9-22	Beef and separable fat/Ground meat	0.2	52.8
9-87f	Pork products/Sausage/Lyoner	0.2	54.2
9-49b	Chicken and separable fat/Thigh/Broiler	0.2	55.5
9-68a	Pork and separable fat/Loin, separable lean/Large-type breeds	0.2	56.8
17-16b	Spices/Curry/Roux	0.2	58.1
8-84a	Mackerel/Raw/Uncooked	0.2	59.3
17-10a	Seasonings/Mayonnaise/Whole egg type	0.2	60.4
9-47b	Chicken and separable fat/Breast, total edible/Broiler	0.2	61.5
9-7b	Beef and separable fat/Chuck loin, total edible/Dairy fattened steer	0.2	62.5

^1^Food codes and descriptions correspond to those of the Standard Tables of Food Composition, 4th revised edition in Japan by Science and Technology Agency.

^2^Data on subjects in Ishikawa PHC (14-day data) were counted twice for 28-day data.

**Table 11.** Cumulative % contribution of the top 20 foods for monounsaturated fatty acid assessed by DR

Food code^1^	Description^1^	g/day	Cumulative^2^ percent
Men (n=102)			
5-15	Vegetable oils/Vegetable oil, mixed	3.5	18.0
10-5a	Chicken egg/Whole egg/fresh	1.7	26.6
11-2	Liquid milk/Ordinary liquid milk	0.9	31.4
9-70a	Pork/Belly without separable fat	0.9	35.9
1-41d	Rice (Paddy rice)/Grains/Well-milled rice	0.7	39.4
17-10a	Seasonings/Mayonnaise/Whole egg type	0.7	42.9
9-13b	Beef/Frank, Short plate, total edible	0.4	45.1
8-95a	Pacific saury/Raw/Uncooked	0.4	47.0
9-49b	Chicken and separable fat/Thigh/Broiler	0.3	48.8
9-47b	Chicken/Breast, total edible/Broiler	0.3	50.5
7-21a	Tofu, soybean curd/Momen	0.3	52.2
8-84a	Mackerel/Raw/Uncooked	0.3	53.9
9-22	Beef and separable fat/Ground meat	0.3	55.5
9-68a	Pork and separable fat/Loin, separable lean	0.3	56.9
9-85a	Pork products/Bacon	0.3	58.3
9-87f	Pork products/Sausage/Lyoner	0.2	59.6
9-7b	Beef/Chuck loin, total edible	0.2	60.9
8-65	Kichiji, rockfish/Raw	0.2	62.0
7-32c	Miso/Rice-koji Miso/Dark yellow type	0.2	63.1
5-7a	Fats and oils/Margarine/Soft type	0.2	64.2
Women (n=113)			
5-15	Vegetable oils/Vegetable oil, mixed	3.0	17.2
10-5a	Chicken egg/Whole egg/fresh	1.4	25.2
11-2	Liquid milk/Ordinary liquid milk	1.1	31.4
9-70a	Pork/Belly without separable fat	0.8	36.1
17-10a	Seasonings/Mayonnaise/Whole egg type	0.7	40.4
1-41d	Rice (Paddy rice)/Grains/Well-milled rice	0.4	42.9
9-49b	Chicken and separable fat/Thigh/Broiler	0.3	44.8
8-95a	Pacific saury/Raw/Uncooked	0.3	46.6
9-13b	Beef/Frank, Short plate, total edible	0.3	48.3
7-21a	Tofu, soybean curd/Momen	0.3	49.9
9-47b	Chicken/Breast, total edible/Broiler	0.3	51.4
9-85a	Pork products/Bacon/Bacon	0.3	52.9
9-87f	Pork products/Sausage/Lyoner	0.3	54.4
8-84a	Mackerel/Raw/Uncooked	0.2	55.8
4-48b	Confectioneries/Doughnut/Cake doughnut	0.2	57.1
9-22	Beef and separable fat/Ground meat	0.2	58.4
9-68a	Pork and separable fat/Loin, separable lean	0.2	59.7
5-7a	Fats and oils/Marganne/Soft type	0.2	60.9
9-7b	Beef/Chuck loin, total edible	0.2	62.0
17-16b	Spices/Curry/Roux	0.2	63.1

^1^Food codes and descriptions correspond to those of the Standard Tables of Food Composition, 4th revised edition in Japan by Science and Technology Agency.

^2^Data on subjects in Ishikawa PHC (14-day data) were counted twice for 28-day data.

**Table 12.** Cumulative % contribution of the top 20 foods for polyunsaturated fatty acid assessed by DR

Food code^1^	Description^1^	g/day	Cumulative^2^ percent
Men (n=102)			
5-15	Vegetable oils/Vegetable oil, mixed	5.2	32.8
1-41d	Rice (Paddy rice)/Grains/Well-milled rice	0.9	38.6
7-21a	Tofu, soybean curd/Momen	0.8	43.9
17-10a	Seasonings/Mayonnaise/Whole egg type	0.6	47.9
7-32c	Miso/Rice-koji Miso/Dark yellow type	0.6	51.8
10-5a	Chicken egg/Whole egg/fresh	0.6	55.7
7-22	Tofu and Abura-age/Okinawa-tofu	0.4	58.4
7-29	Natto, fermented soybeans/Itohiki-natto	0.4	60.8
7-25	Tofu and Abura-age/Abura-age	0.4	63.3
8-153c	Tuna/Canned with Oil; solids and liquid	0.3	64.9
8-84a	Mackerel/Raw/Uncooked	0.3	66.5
9-70a	Pork/Belly without separable fat	0.2	67.8
8-95a	Pacific saury/Raw/Uncooked	0.2	69.1
7-27	Tofu and Abura-age/Kori-dofu	0.2	70.2
5-7a	Fats and oils/Margarine/Soft type	0.2	71.3
1-13a	Wheat (Breads)/White bread/On the market	0.1	72.1
9-49b	Chicken and separable fat/Thigh/Broiler	0.1	72.9
9-47b	Chicken/Breast, total edible/Broiler	0.1	73.7
17-9a	Seasonings/Salad dressings/Separable type	0.1	74.4
6-9	Nuts and Seeds/Walnuts, roasted	0.1	75.1
Women (n=113)			
5-15	Vegetable oils/Vegetable oil, mixed	4.4	32.0
17-10a	Seasonings/Mayonnaise/Whole egg type	0.7	37.0
7-21a	Tofu and Abura-age/Okinawa-tofu	0.7	42.0
1-41d	Rice (Paddy rice)/Grains/Well-milled rice	0.6	46.3
7-32c	Miso/Rice-koji Miso/Dark yellow type	0.5	50.1
10-5a	Chicken egg/Whole egg/fresh	0.5	53.8
7-25	Tofu and Abura-age/Abura-age	0.4	56.5
7-29	Natto, fermented soybeans/Itohiki-natto	0.4	59.1
7-22	Tofu and Abura-age/Okinawa-tofu	0.3	61.5
8-153c	Tuna/Canned with Oil; solids and liquid	0.2	63.1
9-70a	Pork/Belly without separable fat	0.2	64.5
8-84a	Mackerel/Raw/Uncooked	0.2	65.8
5-7a	Fats and oils/Margarine/Soft type	0.2	67.2
8-95a	Pacific saury/Raw/Uncooked	0.2	68.4
1-13a	Wheat (Breads)/White bread/On the market	0.2	69.5
7-27	Tofu and Abura-age/Kori-dofu	0.2	70.7
17-9a	Seasonings/Salad dressings/Separable type	0.1	71.6
6-9	Nuts and Seeds/Walnuts, roasted	0.1	72.6
6-12b	Nuts and Seeds/Sesame seeds/Roasted	0.1	73.5
11-2	Liquid milk/Ordinary liquid milk	0.1	74.3

^1^Food codes and descriptions correspond to those of the Standard Tables of Food Composition, 4th revised edition in Japan by Science and Technology Agency.

^2^Data on subjects in Ishikawa PHC (14-day data) were counted twice for 28-day data.

**Table 13.** Cumulative % contribution of the top 20 foods for n-3 polyunsaturated fatty acid assessed by DR

Food code^1^	Description^1^	g/day	Cumulative^2^ percent
Men (n=102)			
5-15	Vegetable oils/Vegetable oil, mixed	0.87	25.2
8-84a	Mackerel/Raw/Uncooked	0.23	31.7
8-95a	Pacific saury/Raw/Uncooked	0.19	37.2
17-10a	Seasonings/Mayonnaise/Whole egg type	0.14	41.2
7-21a	Tofu, soybean curd/Momen	0.10	44.1
7-32c	Miso/Rice-koji Miso/Dark yellow type	0.10	46.9
8-26a	Sardines/Japanese pilchard/Raw	0.08	49.3
8-77	Salmon/Chum salmon, raw	0.08	51.7
8-78a	Mild salted chum salmon/Uncooked	0.08	54.0
10-5a	Chicken egg/Whole egg/fresh	0.08	56.2
8-65	Kichiji, rockfish/Raw	0.07	58.3
8-81	Salmon/Salted roe	0.07	60.5
8-142	Yellowtail/Cultured, young, raw	0.07	62.5
7-29	Natto, fermented soybeans/Itohiki-natto	0.07	64.4
8-69	Sablefish/Raw	0.06	66.0
7-25	Tofu and Abura-age/Abura-age	0.05	67.4
7-22	Tofu and Abura-age/Okinawa-tofu	0.05	68.7
8-85b	Mackerel/Salted/Mild salted and semi-dried	0.05	70.1
8-124	Herring/Raw	0.04	71.3
8-41	Eel/Kabayaki	0.04	72.4
Women (n=113)			
5-15	Vegetable oils/Vegetable oil, mixed	0.75	25.0
8-84a	Mackerel/Raw/Uncooked	0.17	30.8
8-95a	Pacific saury/Raw/Uncooked	0.16	36.0
17-10a	Seasonings/Mayonnaise/Whole egg type	0.16	41.3
7-32c	Miso/Rice-koji Miso/Dark yellow type	0.08	44.0
7-21a	Tofu, soybean curd/Momen	0.08	46.8
8-77	Salmon/Chum salmon, raw	0.07	49.3
8-78a	Mild salted chum salmon/Uncooked	0.07	51.7
8-26a	Sardines/Japanese pilchard/Raw	0.06	53.8
10-5a	Chicken egg/Whole egg/fresh	0.06	56.0
7-29	Natto, fermented soybeans/Itohiki-natto	0.06	58.1
8-81	Salmon/Salted roe	0.06	60.1
8-65	Kichiji, rockfish/Raw	0.06	62.1
8-142	Yellowtail/Cultured, young, raw	0.05	63.9
7-25	Tofu and Abura-age/Abura-age	0.05	65.4
8-85b	Mackerel/Salted/Mild salted and semi-dried	0.05	67.0
8-69	Sablefish/Raw	0.04	68.2
7-22	Tofu and Abura-age/Okinawa-tofu	0.04	69.5
1-13a	Wheat (Breads)/White bread/On the market	0.03	70.6
8-124	Herring/Raw	0.03	71.6

^1^Food codes and descriptions correspond to those of the Standard Tables of Food Composition, 4th revised edition in Japan by Science and Technology Agency.

^2^Data on subjects in Ishikawa PHC (14-day data) were counted twice for 28-day data.

**Table 14.** Cumulative % contribution of the top 20 foods for n-6 polyunsaturated fatty acid assessed by DR

Food code^1^	Description^1^	g/day	Cumulative^2^ percent
Men (n=102)			
5-15	Vegetable oils/Vegetable oil, mixed	4.32	35.2
1-41d	Rice (Paddy rice)/Grains/Well-milled rice	0.92	42.6
7-21a	Tofu, soybean curd/Momen	0.74	48.6
10-5a	Chicken egg/Whole egg/fresh	0.53	53.0
7-32c	Miso/Rice-koji Miso/Dark yellow type	0.53	57.3
17-10a	Seasonings/Mayonnaise/Whole egg type	0.49	61.3
7-22	Tofu and Abura-age/Okinawa-tofu	0.36	64.2
7-25	Tofu and Abura-age/Abura-age	0.34	67.0
7-29	Natto, fermented soybeans/Itohiki-natto	0.32	69.6
8-153c	Tuna/Canned with Oil; solids and liquid	0.24	71.6
9-70a	Pork/Belly without separable fat	0.20	73.2
5-7a	Fats and oils/Margarine/Soft type	0.16	74.5
7-27	Tofu and Abura-age/Kori-dofu	0.16	75.8
9-49b	Chicken and separable fat/Thigh/Broiler	0.11	76.6
9-47b	Chicken/Breast,total edible/Bmiler	0.10	77.5
11-2	Liquid milk/Ordinary liquid milk	0.10	78.3
1-13a	Wheat (Breads)/White bread/On the market	0.10	79.1
6-12b	Nuts and Seeds/Sesame seeds/Roasted	0.10	79.9
6-9	Nuts and Seeds/Walnuts,roasted	0.09	80.7
17-9a	Salad dressings/Separable type	0.09	81.4
Women (n=113)			
5-15	Vegetable oils/Vegetable oil, mixed	3.69	34.1
7-21a	Tofu, soybean curd/Momen	0.61	39.8
1-41d	Rice (Paddy rice)/Grains/Well-milled rice	0.59	45.2
17-10a	Seasonings/Mayonnaise/Whole egg type	0.54	50.2
7-32c	Miso/Rice-koji Miso/Dark yellow type	0.45	54.3
10-5a	Chicken egg/Whole egg/fresh	0.45	58.5
7-25	Tofu and Abura-age/Abura-age	0.33	61.5
7-29	Natto, fermented soybeans/Itohiki-natto	0.29	64.2
7-22	Tofu and Abura-age/Okinawa-tofu	0.28	66.9
8-153c	Tuna/Canned with Oil; solids and liquid	0.21	68.8
9-70a	Pork/Belly without separable fat	0.18	70.5
5-7a	Fats and oils/Margarine/Soft type	0.17	72.1
7-27	Tofu and Abura-age/Kori-dofu	0.14	73.4
6-12b	Nuts and Seeds/Sesame seeds/Roasted	0.13	74.5
1-13a	Wheat (Breads)/White bread/On the market	0.12	75.7
11-2	Liquid milk/Ordinary liquid milk	0.12	76.8
6-9	Nuts and Seeds/Walnuts, roasted	0.11	77.8
17-9a	Seasonings/Salad dressings/Separable type	0.10	78.7
9-49b	Chicken and separable fat/Thigh/Broiler	0.10	79.6
9-47b	Chicken/Breast,total edible/Broiler	0.08	80.4

^1^Food codes and descriptions correspond to those of the Standard Tables of Food Composition, 4th revised edition in Japan by Science and Technology Agency.

^2^Data on subjects in Ishikawa PHC (14-day data) were counted twice for 28-day data.

## DISCUSSION

In the present study, we examined the correlation between dietary fatty acid intakes estimated from FFQ and DR, and the serum phospholipid fatty acid level to evaluate the FFQ used in JPHC Study Cohort I. Absolute intakes of fatty acid estimated from FFQ were slightly higher than intakes from DR for SFA and MUFA, although no apparent difference was observed for PUFA; on the contrary, intakes from FFQ were lower than those from DR for marine-origin n-3 PUFA both in either men or women.

The highest correlations were observed between the intakes estimated from FFQ and DR for marine-origin n-3 PUFA both in men and women. It is reasonable to think that marine-origin n-3 PUFA intakes are relatively easy to estimate by FFQ because dietary fish greatly contributed to marine-origin n-3 PUFA such as EPA, docosapentaenoic acid (DPA), and DHA. When intake was expressed as a percentage of total fatty acid intake, the correlation was higher than expressed as the absolute intake (g/day) for the marine-origin n-3 PUFA. In contrast, as for SFA and MUFA, intake expressed as an energy-adjusted value was more closely correlated. This is consistent with observations from other studies, and the observed correlation coefficients in the present study were similar to those of other studies for SFA (0.54-0.72) and MUFA (0.49-0.56).^11^^-^^15^ No significant correlation was observed for n-6 PUFA because its contribution was largely by cooking oil. (We did not inquire as to the kind of cooking oil in either the DR or FFQ.) In addition, n-6 PUFA contributed by many kinds of lean foods such as rice, miso, and tofu. Because these lean foods were consumed on a daily basis and might have a small between-person variability, the observed correlation coefficient was likely to attenuate.

Our study revealed a significant correlation between dietary intake of marine-origin n-3 PUFA estimated from both FFQ and DR and the corresponding FA composition of serum phospholipid. The correlation between EPA intake and the corresponding EPA composition of serum phospholipid was much higher estimated from DR than from FFQ. No apparent differences in correlations were observed for other marine-origin n-3 PUFA. The correlation observed for EPA not only estimated from DR but also from FFQ was higher than the correlation coefficient of 0.20-0.57 observed in the previous studies for the correlation between dietary intake of EPA and EPA content in biological specimens such as adipose tissue, serum, and plasma.^16^^-^^20^ The correlations observed for DHA were similar to those reported in several studies (0.42-0.56).^17^^-^^20^ Just as with the correlation between the intakes estimated from FFQ and from DR, when intake was expressed as a percentage of total fatty acid intake, the correlation was higher than expressed as the absolute intake (g/day) for the marine-origin n-3 PUFA. This is consistent with other reports.^16^^,^^18^^-^^20^ The previous study reported that the marine-origin n-3 PUFA was less important for the smaller contributors to intake, since they were much less correlated with total fatty acid intake.^16^ We also observed a significant correlation between dietary intake of MUFA estimated from DR (expressed as percentage of total fatty acid intake) and the corresponding FA in serum phospholipid. In the previous study, the observed correlation between MUFA intake and those of biomarker was weak,^16^^-^^20^ possibly because of the endogenous synthesis of MUFA from carbohydrate and the relatively large within-person variability and small between-person variability of oleic acid intake.^11^^,^^18^ It is expected that olive oil intake, the source of oleic acid, would be small, and the contribution of eggs or dairy product consumption to the MUFA would be relatively high in our study population.

In summary, we observed a relatively high correlation between fatty acid intake estimated from FFQ and intake estimated from DR for SFA and MUFA when expressed as an energy-adjusted value, and for marine-origin n-3 PUFA when expressed as a percentage of total fatty acid intake. We also observed a relatively high correlation between fatty acid intake estimated from FFQ and serum phospholipid fatty acid level for marine-origin n-3 PUFA. As for the marine-origin n-3 PUFA, the correlation was relatively high as expressed in all 3 ways, although when expressed as a percentage of total fatty acid intake the correlation was highest. These results suggested that when using the data of fatty acid intake assessed with FFQ for JPHC study, we should take into account the indicator of the intakes of each kind of fatty acid.
